# Prevalence of and Factors Associated With Depression and Anxiety Among Job‐Seeking Graduates: A Cross‐Sectional Study on Bangladesh

**DOI:** 10.1002/puh2.70279

**Published:** 2026-05-27

**Authors:** Ashraful Alam, Abdullah Al Marin, Abdullah Al Zubayer, MD. Sameer Hasan, Md. Shahria Ahmed Arzu, Lisa Sharma, Faria Islam Mim, Injamamul Haque, Sanwaya Palit, Fayeza Tahia Rahman, Anna Annie Gomes, Md. Ibrahim Azad, Raisa Imran Chowdhury, Safayet Jamil, Mohammad Shahangir Biswas

**Affiliations:** ^1^ Institute of Forestry & Environmental Sciences University of Chittagong Chattogram Bangladesh; ^2^ Economics Discipline Khulna University Khulna Bangladesh; ^3^ Department of Sociology University of Barishal Barishal Bangladesh; ^4^ Department of Marketing Faculty of Business Administration Patuakhali Science & Technology University, Dumki Patuakhali Bangladesh; ^5^ Department of Economics University of Rajshahi Rajshahi Bangladesh; ^6^ BRAC Institute of Governance & Development BRAC University Dhaka Bangladesh; ^7^ Department of Sociology Eden Mohila College National University Gazipur Bangladesh; ^8^ Faculty of Business Administration Cox's Bazar International University Cox's Bazar Bangladesh; ^9^ Popular Medical College, Dhanmondi Dhaka Bangladesh; ^10^ Department of Economics Southeast University Dhaka Bangladesh; ^11^ Department of Journalism and Media Communication Green University of Bangladesh Dhaka Bangladesh; ^12^ Institute of Disaster Management and Vulnerability Studies (IDMVS) University of Dhaka Dhaka Bangladesh; ^13^ Department of Public Health Daffodil International University Dhaka Bangladesh; ^14^ Department of Biochemistry and Biotechnology University of Science and Technology Chittagong Chattogram Bangladesh

**Keywords:** anxiety, Bangladesh, depression, factors, graduate, jobseekers

## Abstract

The purpose of this cross‐sectional study was to determine the prevalence and factors associated with depression and anxiety among job‐seeking graduates in Bangladesh, as mental health issues are often overlooked or stigmatized. The 689 participants were selected by means of convenience sampling method. A self‐reported questionnaire was used to gather data with sociodemographic data, lifestyle characteristics, 7‐item generalized anxiety disorder scale (GAD‐7), and Patient Health Questionnaire‐9 (PHQ‐9). It was established that 48.5% of the study population experienced depression and anxiety at 39.3%. Approximately 86% of individuals who reported the presence of depression and 87.8% of individuals who reported anxiety had finished studying back in the previous 2 years. Multivariable logistic regression models demonstrated that education level was found to be significantly linked with depression (adjusted odds ratio [aOR] = 1.35; 95% confidence interval [CI] = 0.908–2.022; *p* < 0.05), as was social media use (aOR = 1.50; 95% CI = 1.089–2.073; *p* < 0.05). Furthermore, gender was significantly associated with anxiety (aOR = 1.57; 95% CI = 1.092–2.255; *p* < 0.05). The study features the psychological vulnerability of job seekers in Bangladesh, emphasizing the social need to recognize unemployment‐related mental health challenges and reduce stigma around seeking support. Findings call for integrating mental health support into employment services and career programs. The study also lays groundwork for further research on mental health interventions targeting unemployed youth. Lastly, the current article releases a scarce perspective of the rates and causes of depression and anxiety in Bangladesh in the light of a new timeframe.

## Graphical abstract

A cross‐sectional study has exposed huge prevalence of depression (48.5) and anxiety (39.3) among the Bangladeshi job‐seeking graduates, where excessive social media engagement has predicted depression, and females have demonstrated more association with anxiety compared to male. Therefore, urgent mental health intervention has become a must for such vulnerable population.

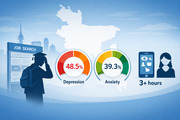

## 1 Introduction

Each circumstance that interferes with an individual's thoughts, emotions, actions, or capacity to carry out daily activities can be described as a mental health disorder. Mental health disorders are at the top 10 of major global burdens of conditions of disease and disability [1] because depression and anxiety disorders are supposed to be the third and ninth leading causes, respectively, behind global disability [2]. Such mental disorders are linked with several negative health outcomes. The comorbidity between mental illnesses in general and chronic disease conditions is high. Studies indicate that there is a strong correlation between depression and chronic disorders, including heart disease and diabetes [3, 4, 5]. Depressed people have a 60% higher chance of developing Type 2 diabetes than people who have not screened positive to depression [3]. Studies also indicate that depression and anxiety are linked to some risky health behaviors like drug abuse and committing suicide [6, 7].

Anxiety and depression constitute the most common mental conditions with a large percentage of the world population being affected. The world has estimated numbers of 264 and 284 million individuals with depression and anxiety, respectively [8]. Though people of all ages experience mental health disorders, young people aged between 20 and 35 years are more vulnerable to these disorders. A study on the United States demonstrated that job insecurity brought about anxiety and depression among young adults aged between 15 and 29 years old [9]. Parallelly, another study revealed that among people of 18–29 years old are most likely to be affected by depression and anxiety [10]. Approximately 29% of the adults are anxious [11] and 29% are depressed at some time in their lives [12].

Although these challenges are a global concern, the burden of mental health problems is also widespread in developing countries like Bangladesh. Depression and anxiety are highly prevalent among Bangladeshi populations. Bangladesh, a country in the South Asian region, has a population of around 170 million, and nearly 28% of the population is between 15 and 29 years old [13]. A past cross‐sectional study conducted in Bangladesh among 311 adolescents (13–17 years old) described that over 36% of the sample subjects managed to report experiencing depressive symptoms according to the 9‐item Patient Health Questionnaire (PHQ‐9) [14]. Another Bangladeshi study assessed university students’ mental health by using the Goldberg's General Health Questionnaire and reported that around 60% of students were found to be having worse mental health [15]. Using the 21‐item Bangla Anxiety, Depression, and Stress Scale, Rabby et al. reported the rate of depression, anxiety, and stress in Bangladesh among students, who study in universities and are asked to complete their entrance admission process as 57.7%, 61.4%, and 44.6%, respectively [16]. In Bangladesh, a national household survey of adults aged 18 years and more, 7270 individuals completed a survey in 2019 in which over 18% of the respondents reported being diagnosed with a mental disorder according to Diagnostic and Statistical Manual of Mental Disorders [17].

University and college graduates are typically young individuals. They aim to achieve economic independence and find employment to support their family after obtaining their desired university degree. At a young age, people are expected to form familial relationships, become financially independent, and take on responsible roles as active and productive members of their families and communities [18]. Significant transitions in life, including career choices, financial difficulties, relationship changes, sensitivity to peer pressure, and pressure to reach goals quickly make young adults vulnerable to mental health disorders [19, 20, 21]. In Bangladesh, there are 172 universities and more than 1000 colleges offering bachelor's and master's degrees [22]. Every year, over 650,000 students graduate from these institutions and enter the job market. However, finding a desired job in Bangladesh can be quite stressful due to the highly competitive nature of the job market, which may negatively impact their mental health. Additionally, lengthy recruitment and selection processes, along with pressure from families to secure employment, further contribute to stress among job‐seeking graduates. Research in the past has indicated that mental illnesses, depression, and anxiety being the most common are common in the Bangladesh population [13, 17, 23]. For instance, a study revealed that among people of 18–29 years old are most likely to be affected by depression and anxiety [10].

Despite there is the rising prevalence of mental health issues among youth population in Bangladesh, the mental wellbeing conditions of graduate job seekers are largely ignored. Nevertheless, the problems have barely been investigated among graduates from the country seeking a job. It might be a burden to the nation in the long run. Therefore, in the light of previous studies, this study sought to examine unstudied connected factors of depression and anxiety among job‐seeking graduates in Bangladesh.

## 2 Materials and Methods

### 2.1 Study Design and Settings

The cross‐sectional research was carried out with the job‐seeking graduates in the Dhaka City, Bangladesh between, June 20 and July 10, 2024. Dhaka is supposed to be a major urban center consisting of 9 public universities, 57 private universities, and more than 50 colleges. So, it is considered a hub for higher education and employment opportunities. Side by side, many graduates migrate or remain in Dhaka for job prospects and relevant preparation as majority of the job examinations and job fairs take place in Dhaka city.

### 2.2 Participants’ Eligibility Criteria

In this study, job‐seeking graduates were those people actively initiating and pursuing actions towards searching for new employment or reemployment including people who were preparing for job exams, applying for jobs, attending job interviews in public or private sectors. Participants were eligible for inclusion in this study if they had completed a bachelor's or master's degree within the past 5 years, were currently seeking employment in either the public or private sector, had been residing in Dhaka City for at least 1 year, and were willing to participate voluntarily. Individuals who had secured any form of employment like full‐time, part‐time, contractual and self‐employed or who had physical or mental impairments that could affect participation were excluded from the study.

### 2.3 Sample Size and Sampling Method

The minimum required sample size for this study was calculated at 384 using the standard formula, $n=\frac{Z^{2}\times p\times \left(1-p\right)}{e^{2}}$ [24], with a 5% margin of error and 95% confidence level, and assuming the prevalence of depression or anxiety of 50%, where *n* = minimum required sample, *Z* = 1.96 for 95% confidence level, *p* = 0.5, assumed prevalence of depression/anxiety, and *e* = 0.05, precision of prevalence estimate. By putting values, we get *n* = {(1.96)^2^ × 0.5 × (1 − 0.5)}/(0.05)^2^ or *n* = (0.9604/0.0025).

So, *n* is the 384.16.

However, a total of 689 participants were finally included. Participants were recruited using a convenience sampling method due to the absence of a sampling frame for job‐seeking graduates in Dhaka city.

### 2.4 Participant Characteristics

This study involving 689 research respondents has adopted some of the important demographic factors, for example, an age, sex, type of education, marital status, residential status, and years since their last degree. These characteristics have not only been applied in the description of the sample but also in the investigation of their relations with depression and anxiety.

### 2.5 Measures

The participants were asked to take a predesigned questionnaire. The following were the four sections of the questionnaire: sociodemographics, lifestyle‐related factors, depression, and anxiety.

#### 2.5.1 Sociodemographic Variables

The sociodemographic variables that were investigated in the study were the age, gender, and the monthly family income of the participants (measured in Bangladeshi taka [BDT]), education level, years since completing education, marital status, and living status.

#### 2.5.2 Lifestyle Related Variables

The lifestyle‐related variables included smoking habits, daily time spent on social media, physical activity, and sleep duration. Social media use was assessed by the question, “During the past 7 days, how much time did you typically spend on social media each day?” Responses of this question were categorized into two groups: “1–3 h/day” and “>3 h/day.” Physical activity was assessed by the question, “During the past 7 days, did you engage in physical activity for at least 60 min/day?” Responses of this question were recorded as “Yes” or “No.” Sleep duration was assessed by the question, “During the past 7 days, how many hours did you sleep on average per night?” Responses of this question were categorized as “≤7 h/night” and “>7 h/night.”

#### 2.5.3 Depression

Participants’ depression was measured using the PHQ‐9 [23, 49]. It has been validated in the context of Bangladesh [25]. Each item was measured on a four‐point Likert scored (“0 = not at all” to “3 = nearly every day”), yielding a total score ranging from 0 to 27. A score of ≥10 on the PHQ‐9 was considered indicative of the presence of depression [25, 26]. This study used the Bengali version of the PHQ‐9. This scale showed a high internal consistency, with a Cronbach's alpha of 0.889 in the study sample.

#### 2.5.4 Anxiety

Participants’ anxiety was measured using the 7‐item generalized anxiety disorder scale (GAD‐7) [27] which has been validated in Bangladesh [28]. Like the PHQ‐9, each item was measured on a four‐point Likert scored (“0 = not at all” to “3 = nearly every day”), yielding a total score ranging from 0 to 21. A score of ≥ 10 was used to determine the presence of anxiety among participants [28, 29]. The Bengali version of the GAD‐7 was used in this study [29]. Cronbach's alpha of the GAD‐7 has been found to be (*α* = 0.89) in the study sample.

### 2.6 Questionnaire Development and Pilot Testing

The questionnaire was formulated in English and then translated to Bengali by two bilingual speakers and pilot tested in terms of clarity and face validity. We conducted the pilot survey of 30 participants from Dhaka checking the inclusion criteria for assessing face validity. It was self‐administered online and mandatory response option was enabled during those surveys for reducing careless responding of the respondents. The participants were selected from different disciplines using convenient sampling who have completed their bachelor's and master's degree. During that process they were asked to comment on the relevance, clarity, and wording of the questions. The feedback we received was used for improving clarity and formatting. Data from the pilot test were not incorporated in the final analysis.

### 2.7 Data Collection Procedure

After obtaining consent from the participants, the final questionnaire that we imported into Google Forms has been distributed via Facebook Messenger, WhatsApp, and email. We have disseminated the corresponding form link through alumni groups, university‐based job preparation groups, and within professional networking groups of such people preparing for public and private jobs. As we provided the link across diverse networks to reach out to participants from different academic and socioeconomic backgrounds, we have no specific probability‐based sampling frame for this study.

### 2.8 Ethical Clearance

The research conducted was based on the Declaration of Helsinki on human research subjects. The participants have been given full details of the research, that is, the aim of the research, what the research involves, what risks and benefits the research may bring, and the privilege of withdrawing at any given time. The study protocol has been accepted by ethical committee of Khwaja Yunis Ali University in Bangladesh (KYAU/DEAN/EGC/2024/12).

As this study did not incorporate any clinical trial or such intervention so no clinical trial registration was needed. Therefore, no clinical trial registration number is applicable for our study.

### 2.9 Data Analysis

Data were analyzed using SPSS software. The dataset prepared in Microsoft Excel format was imported into SPSS for statistical analyses. Descriptive statistics were used to summarize the study variables, such as mean and standard deviation for continuous variables, whereas frequency and percentage for categorical variables. Binary logistic regression analysis was performed to determine the factors associated with depression and anxiety. At first, crude model was performed, to determine the unadjusted associations. Then, we fitted the adjusted model, where we interred variables whose associated *p* values were found to be ≤0.25 in the crude models. Results of the regression analyses were reported as odds ratio (OR) with 95% confidence interval (CI) and associated *p* value. All covariates incorporated in the multivariable model were selected in the light of theoretical relevance from previous literature. Multicollinearity was assessed using variance inflation factor (VIF), which indicated that all the independent variables were not highly correlated as all the values were less than 2. Model fitness was assessed by Hosmer–Lameoshow test, and we found the associated *p* value as 0.759 (for depression) and 0.666 (for anxiety) for the adjusted model, which indicated our model was a good fit. We considered age as a continuous variable, and linearity was assessed and confirmed in this study. Mandatory response option was applied in the online form to have no missing values for key variables so that a complete case analysis could be performed. The relations between variables were considered statistically significant if the *p* value was ≤0.05. However, with relatively high prevalence of anxiety and depression, OR should be interpreted carefully as for common outcome, odd ratio might overestimate the relative risk.

## 3 Results

In the current research no less than 689 participants were involved. The participants’ mean (±SD) age was 25.71 (±1.98) years.

Of the 689 participants, male was 54%, and female was 46%. Approximately 48.6% of the participants reported a family monthly income of 30,000 BDT or less, whereas 51.4% of the participants’ monthly family income was greater than 30,000 BDT. Most participants (73.6%) completed their bachelor's degree, whereas 26.4% held a master's degree. The majority (87.1%) had finished their education within the last 2 years, with only 12.9% having completed their studies more than 2 years ago. Most participants (75.6%) were unmarried. More than half of the participants (58.5%) lived with their families, whereas 41.5% lived apart from them. The majority (74.0%) were nonsmokers, whereas 26.0% reported that they smoked. In terms of social media usage, 49.3% of participants used it for 1–3 h/day, whereas 50.7% used it for more than 3 h/day. Most participants (65.5%) engaged in physical activity for at least 60 min daily, whereas 34.5% did not meet this level of activity. Additionally, 87.1% reported sleeping for 7 h or less per night, whereas only 12.9% slept for more than 7 h (Table 1).

**TABLE 1:** Descriptive characteristics of the study participants.

Variable	Category	*n*/Mean	%/SD
Total	—	689	100.0
Age (in years)	—	25.71	1.98
Gender	Male	372	54.0
	Female	317	46.0
Monthly family income	≤30,000 BDT	335	48.6
	>30,000 BDT	354	51.4
Completed education level	Bachelor's	507	73.6
	Master's	182	26.4
Years since completing education	≤2 years	600	87.1
	>2 years	89	12.9
Marital status	Unmarried	502	75.6
	Married	162, 187	24.4
Living status	With family	403	58.5
	Apart from family	286	41.5
Smoking habit	Yes	179	26.0
	No	510	74.0
Social media use	1–3 h/day	340	49.3
	>3 h/day	349	50.7
Physical activity for at least 60 min a day	No	238	34.5
	Yes	451	65.5
Sleep duration at night	≤7 h	600	87.1
	>7 h	89	12.9
Depression	No	355	51.5
	Yes	334	48.5
Anxiety	No	418	60.7
	Yes	271	39.3

Abbreviations: BDT, Bangladeshi taka; SD, standard deviation.

**FIGURE 1:**
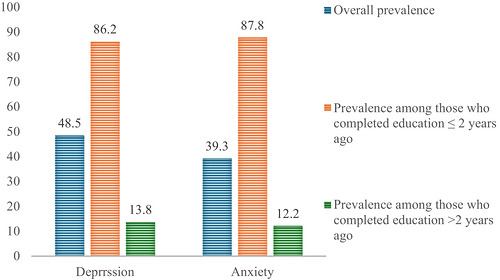
Prevalence of depression and anxiety by years after education completed.

The prevalence of depression among the study participants was 48.5%, as determined by the PHQ‐9 [23]. Among those reporting depression, 86.2% had completed their education, either at the bachelor's or master's level, within the last 2 years, whereas 13.7% had completed their education more than 2 years ago. Anxiety was reported by 39.3% of the participants, as assessed by the GAD‐7 [27]. Among the anxious people, 87.8% of the cases were educated in the recent 2 years, and 12.2% were educated more than 2 years ago (Figure 1).

In the adjusted regression model social media demonstrated significant association with depression as such participants who used social media more than 3 h regularly had 1.48 times higher odds of depression compared to those used social media withing 1–3 h daily (aOR = 1.48, 95% CI = 1.08–2.03, *p* < 0.05). Other variables, such as age, gender, family income per month, education, number of years since completion of education, marital status, living arrangement, smoking, physical activity, and sleeping hours, were not significantly associated with depression (*p* > 0.05). Though education level and social media were found significant in the crude model, the associations were weakened after adjustments, implying potential confounding effects (Table 2).

**TABLE 2:** Factors associated with depression among job‐seeking graduates.

Variables	cOR	(95% CI)	*p* value	aOR	(95% CI)
Age	1.06	(0.98–1.14)	0.155	1.01	0.92–1.11
Gender					
Male	Ref				
Female	0.93	(0.69–1.25)	0.610		
Monthly family income					
≤30,000 BDT	1.14	(0.84–1.54)	0.393		
>30,000 BDT	Ref				
Completed education level					
Bachelor's	Ref			Ref	
Master's	0.71	(0.50–0.99)^a^	0.042	1.35	0.91–2.01
Years since completing education					
≤2 years	Ref				
>2 years	0.86	(0.55–1.35)	0.516		
Marital status					
Unmarried	1.28	(0.89–1.82)	0.183	1.24	0.86–1.80
Married	Ref			Ref	
Living status					
With family	Ref			Ref	
Apart from family	0.92	(0.68–1.25)	0.603	0.91	(0.65–1.27)
Smoking habit					
Yes	1.36	(0.97–1.92)			
No	Ref				
Social media use					
1–3 h/day	Ref			Ref	
>3 h/day	0.73	(0.54–0.98)^a^	0.035	1.48	(1.08–2.03)^a^
Physical activity for at least 60 min a day					
No	Ref			Ref	
Yes	1.25	(0.91–1.71)	0.167	0.89	(0.641–1.24)
Sleep duration at night					
≤7 h	Ref			Ref	
>7 h	1.31	(0.83–2.05)	0.243	0.71	(0.44–1.13)

Abbreviations: aOR, adjusted odds ratio; BDT, Bangladeshi taka; CI, confidence interval; cOR, crude odds ratio.

^a^ Significant association at alpha level 0.05.

In this adjusted logistic regression model, gender and sleep duration were significantly associated with anxiety. The outcome demonstrated that female respondents had 1.39 times higher odds than male (aOR = 0.57, 95% CI = 0.35–0.95, *p* < 0.05). In addition, respondents who slept more than 7 h daily had 43% lower odds in comparison to those sleeping ≤7 h daily (aOR = 1.39, 95% CI = 1.00–1.94, *p* < 0.05). Other variables, such as age, family income per month, education, number of years since completion of education, marital status, living arrangement, smoking, physical activity, and social media usage, were not significantly associated with anxiety (*p* > 0.05) (Table 3).

**TABLE 3:** Factors associated with anxiety among job‐seeking graduates.

Variables	cOR	*p* value	95% CI	aOR	95% CI
Age	1.06	0.147	(0.98–1.14)	1.01	(0.92–1.11)
Gender					
Male	Ref			Ref	
Female	0.70	0.025	(0.52–0.96)^a^	1.39	(1.00–1.94)^a^
Monthly family income					
≤30,000 BDT	0.89	0.457	(0.66–1.21)		
>30,000 BDT	Ref				
Completed education level					
Bachelor's	Ref			Ref	
Master's	0.73	0.066	(0.51–1.02)	1.17	(0.78–1.75)
Years since completing education					
≤2 years	Ref				
>2 years	1.12	0.641	(0.70–1.77)		
Marital status					
Unmarried	0.93	0.706	(0.65–1.34)	0.99	(0.67–1.47)
Married	Ref			Ref	
Living status					
With family	Ref				
Apart from family	1.06	0.639	(0.78–1.45)		
Smoking habit					
Yes	1.31	0.127	(0.93–1.85)		
No	Ref				
Social media use					
1–3 h/day	Ref			Ref	
>3 h/day	0.81	0.173	(0.60–1.12)	1.32	(0.95–1.83)
Physical activity for at least 60 min a day					
No	Ref			Ref	
Yes	1.32	0.089	(0.96–1.82)	0.81	(0.58–1.14)
Sleep duration at night					
≤7 h	Ref			Ref	
>7 h	1.574	0.064	(0.97–2.55)	0.57	(0.35–0.95)^a^

Abbreviations: aOR, adjusted odds ratio; BDT, Bangladeshi taka; CI, confidence interval; cOR, crude odds ratio.

^a^ Significant association at alpha level 0.05.

## 4 Discussion

The findings revealed that the prevalence rates of depression and anxiety among the study participants were 48.5% and 39.3%, respectively. These rates were quite a bit higher than those reported in previous studies involving young adults. For instance, using the similar questionnaires (i.e., the PHQ‐9) [17] and the GAD‐7 [27], a previous study conducted among dental university students in Bangladesh reported prevalence rates of depression and anxiety at 27.4% and 18.2%, respectively [30]. Additionally, another study found that the prevalence of anxiety (based on the GAD‐7) [27] among young people aged 16–28 years was 31.2% in Bangladesh [31]. These comparisons highlight an increased trend of mental health issues among job‐seeking graduates in Bangladesh.

Job‐seeking graduates often experience a significant burden of depression and anxiety. Several factors, including pressure to find a job quickly, the uncertainty and competitiveness of the job market, financial concerns, and the perceived expectation of immediate success after graduation, could all contribute to significant stress and mental health challenges among these populations during the job‐seeking period.

This study further found that among participants reporting depression, 86.2% had finished their education in 2 years or less. And 87.8% of those experiencing anxiety had completed their education within the same 2‐year timeframe. This means that actively employed graduates tend to acquire ill mental health within the first 2 years of their education like depression and anxiety. This may be because they expect more opportunities of getting a job once they graduate. Inability to fulfill this expectation may cause depression and anxiety.

This work concluded that individuals who have already received their master's degree were much more predisposed toward depression than those having a bachelor's degree. In Bangladesh, a master's degree is assumed to be the highest degree in education, and the participants with this degree are expected to acquire a job soon after they have pursued the degree. Failure of which may add to the occurrence of depression within them. This study demonstrated that participants who used social media more than 3 h a day tended to be depressed compared to participants who used the media 3 h or less. This observation was agreeable to the literature of the past years [32, 33, 34], that reported that depression was associated with overuse of social media. Within social media application platforms like Facebook and Instagram, people see perfectly selected and perfect lives. This would make them contrast those lives with their own and this may also cause depression. However, reverse causality could not be ruled out for the cross‐sectional nature of study. It might be equally plausible that people with depression might spend more time on social media as a coping mechanism or because of less interest in offline daily life courses. So, this particular outcome should not be considered causal. On the anxiety measure, the research resulted in the fact that the respondents were more prone to anxiety than their male counterparts. The same was consistent with other studies [21, 35]. Females typically have higher burden of mental health issues [36], like anxiety than males due to different coping strategies and different societal expectations [37, 38].

These outcomes could also be interpreted in the light of established theoretical frameworks like “social comparison theory,” which implies that individuals compare their lives with the curated and frequently idealized representations visualized on social media for assessing themselves. Considering job‐seeking graduates, exposure to peers’ accomplishments or career achievements might increase their feelings of insufficiency, resulting in elevated symptoms of depression or anxiety [39, 40]. Besides, “life transition theory” demonstrated that the immediate after graduation phase of life might be a critical life transition due to uncertainty, delicate expectations, and everchanging social roles [41, 42]. Such transitional strains might exacerbate psychological hardship, remarkably among graduates who experienced lingering job searches and financial burdens. Due to the cross‐sectional nature, in this study, we tried to identify associations other than casual pathways, and integration of these theoretical perspectives might help strengthen the associations we found.

### 4.1 Limitations

Our study got several considerable limitations while interpreting the results. First, due to the cross‐sectional nature of the study, causal inference or causal relationships between variables had not been provided. Besides, for the same reason, this study lacked determining the temporal direction between depression and social media usage. Second, applying convenience sampling technique and social media‐based recruitment might bring about recall bias and selection bias as well. People who were more involved with social media and job preparation groups might have responded more, which perhaps influenced prevalence estimation and measured associations as well. So, our outcomes might not be completely representative of all the job‐seeking graduates from Bangladesh. Third, collecting data online could exclude graduates with inadequate internet access. For such issues, generalizability of this study might be limited.

## 5 Conclusion

This study highlighted the current circumstances of depression and anxiety among the job‐seeking graduates of Bangladesh. Besides, it indicated a critical need for mental health attention as approximately 50% of the sample reported perceiving depression and about 40% reported perceiving anxiety; this shows that there is a sizeable amount of job‐seeking graduates in Bangladesh who are facing these mental health problems. Our findings figured out that psychological distress had been commonly found in the early postgraduation period. Specifically, a vulnerable phase of young adults was noticed during the transition from education to employment. To combat this by policy, employment and youth development programs can include mental health testing and basic necessary services as per job placement schemes. For graduates, government and nongovernment organizations can consider incorporating psychological support systems into career development programs and employment training centers. Besides, universities’ authorities can arrange career guidance programs by facilitating students with various workshops focusing on stress management, mental resilience training, and so on. Moreover, graduate support systems can be made to support students during their job‐seeking process. Lastly, public awareness campaigns should be expanded to improve mental health literacy among youth.

## Author Contributions

**Injamamul Haque**: methodology, validation, writing – review and editing, writing – original draft, software. **Sanwaya Palit**: conceptualization, writing – original draft, methodology. **Ashraful Alam**: conceptualization, methodology, writing – review and editing, validation, formal analysis, data curation, supervision. **Abdullah Al Zubayer**: validation, conceptualization, supervision, project administration, methodology. **Anna Annie Gomes**: data curation, visualization, writing – original draft, writing – review and editing. **Abdullah Al Marin**: conceptualization, methodology, software, formal analysis, writing – original draft, writing – review and editing. **Raisa Imran Chowdhury**: formal analysis, conceptualization, writing – original draft. **Fayeza Tahia Rahman**: writing – original draft, validation, data curation. **Lisa Sharma**: visualization, methodology, writing – review and editing, writing – original draft, data curation. **Faria Islam Mim**: visualization, methodology, writing – review and editing, writing – original draft, data curation. **Safayet Jamil**: conceptualization, validation, writing – original draft. **Mohammad Shahangir Biswas**: writing – review and editing, validation, supervision. **MD. Sameer Hasan:** data curation, methodology, validation, writing – review & editing, writing – original draft, conceptualization. **Md. Shahria Ahmed Arzu:** writing – original draft, writing – review & editing, methodology, data curation, onceptualization. **Md. Ibrahim Azad**: software, writing – original draft, validation

## Funding

The authors have nothing to report.

## Conflicts of Interest

The authors declare no conflicts of interest.

## Transparency Statement

The authors affirm that this manuscript is an honest, accurate, and transparent account of the study being reported; that no important aspects of the study have been omitted; and that any discrepancies from the study as planned (and, if relevant, registered) have been explained.

## Data Availability

Data will be available on request.
